# Electrochemistry in supercritical fluids

**DOI:** 10.1098/rsta.2015.0007

**Published:** 2015-12-28

**Authors:** Jack A. Branch, Philip N. Bartlett

**Affiliations:** Department of Chemistry, University of Southampton, Southampton SO17 1BJ, UK

**Keywords:** electrochemistry, supercritical fluid, electrodeposition, metallocene

## Abstract

A wide range of supercritical fluids (SCFs) have been studied as solvents for electrochemistry with carbon dioxide and hydrofluorocarbons (HFCs) being the most extensively studied. Recent advances have shown that it is possible to get well-resolved voltammetry in SCFs by suitable choice of the conditions and the electrolyte. In this review, we discuss the voltammetry obtained in these systems, studies of the double-layer capacitance, work on the electrodeposition of metals into high aspect ratio nanopores and the use of metallocenes as redox probes and standards in both supercritical carbon dioxide–acetonitrile and supercritical HFCs.

## 1. Introduction

The supercritical phase was first discovered by Baron Charles Cagniard de la Tour in 1822. Cagniard de la Tour’s first experiment in which he observed the supercritical phase involved rolling a flint ball in a sealed Papin digester (a predecessor to the autoclave) filled with liquid (water, alcohol, ether and carbon disulfide) [1–3]. Rolling the device resulted in a splashing noise as the flint penetrated the liquid–vapour interface [3]. Cagniard de la Tour noted that, by heating the device to far beyond the point of the liquid, the splashing sound stopped. At this point, the boundary between the liquid and gaseous phase was gone, and marked the discovery of the supercritical phase. Cagniard de la Tour also described how he heated a sealed glass tube of alcohol under pressure [1,3]. He observed that the liquid expanded to twice its original volume until it vanished altogether, making the tube appear completely clear [3]. When the system was re-cooled, a thick cloud appeared. Cagniard de la Tour named his discovery ‘état particulier’ [1–3]. He also measured the critical temperature at which the interface tension vanished and discovered that at certain temperatures beyond which total vaporization of the liquid occurs no increase in pressure will liquefy the gas [2,3].

In 1845, Michael Faraday [4] wrote and commented on the critical point; he referred to this as ‘Cagniard de la Tour’s state’ and ‘the Cagniard de la Tour point’. Later in 1861, Dmitri Mendeleev also commented on the phenomenon and referred to it as the ‘absolute Siedetemperatur’ or absolute boiling point [3,5]. Eventually, Thomas Andrews coined the modern term *critical point*[3,6].

In 1869, Andrews [6] described his experiments on the effects of temperature and pressure on sealed glass tubes of partially liquefied carbonic acid. In his paper, he describes the critical point as the temperature was raised to 88°F (304 K) ‘the surface of demarcation between the liquid and gas became fainter, lost its curvature, and at last disappeared’ [6], pp. 575–576. Andrews further noted that the space was occupied by a ‘homogenous fluid, which exhibited, when the pressure was suddenly diminished or the temperature slightly lowered, a peculiar appearance of moving or flickering striae throughout its entire mass’. Andrews noted that the ‘great changes of density which occur about this point (critical point) produce the flickering movements’. Andrews also commented on attempting to take the substance above its critical temperature and pressure; ‘at temperatures above 88°F no apparent liquefaction of carbonic acid, or separation into two distinct forms of matter, could be effected, even when a pressure of 300 or 400 atmospheres was applied’ [6], p. 576.

A substance above its critical temperature (*T*_C_) and pressure (*p*_C_) is called a supercritical fluid (SCF). SCFs have increased transport properties which are a result of their low viscosity [7]. The solvent properties of an SCF can be dramatically altered with only modest changes to temperature or pressure, making them attractive as tuneable solvation media [7]. The gas-like properties of SCFs include low viscosities and the absence of surface tension, while their liquid-like properties allow for the dissolution and transport of materials. SCFs have been applied to fine and speciality chemistry, polymer and material modification, biotechnology and pharmaceuticals, dry cleaning [8], extraction and chromatography [9], and the processing of nanomaterials and nanostructures [10]. SCFs have been employed in making nanowires [11], nanostructures [12], nanocrystalline materials [13], thin film deposition [14] and deposition into mesoporous silica templates [8]. A review by Romang & Watkins [10] highlights the advantages of SCFs for the fabrication of semi-conductor devices.

## 2. Supercritical fluids as solvents for electrochemistry

### (a) Supercritical fluids studied for electrochemistry

Although SCFs have unique properties, including low viscosity that leads to enhanced mass transport to and from the electrode surface [7], there is relatively little literature on SCFs as solvents for electrochemistry. A range of different substances have been used as SCFs for electrochemistry, including ammonia (NH_3_), sulfur dioxide (SO_2_), acetonitrile (CH_3_CN), water (H_2_O) [15] and more recently hydrofluorocarbons (HFCs). HFCs are useful SCFs due to their easily accessible critical temperatures and pressures, high polarity [16] and very high densities in the supercritical state [17]. One of the earliest reports of different SCFs being used for electrochemical processes was by Silvestri *et al.* [18] in 1981. They investigated CO_2_, bromotrifluoromethane (CBrF_3_), hydrogen chloride (HCl) and NH_3_. Their research showed that CO_2_ was a poor conductor in both the liquid and supercritical state [18]. They also noted that CBrF_3_ was a very poor conductor and that electrolytes were practically insoluble; they concluded that both CO_2_ and CBrF_3_ were unsuitable as solvents. Work with scHCl, although having a higher dielectric constant and appreciable solvent properties in the supercritical state, was unsuitable due to its high corrosivity. Even though it was unsuitable for further experiments, elemental iodine was produced at the anode in a scHCl/KI system [18]. Ammonia was also noted to have a higher dielectric and appreciable solvent properties in supercritical conditions; these investigations accomplished the anodic dissolution of silver and iron [18,19].

Bard and co-workers provided the most extensive work on the use of SCFs as solvents for electrochemistry by performing voltammetric studies at near- and supercritical conditions. Bard’s group studied a range of inorganic compounds in a range of supercritical solvents; these solvents included NH_3_, H_2_O, CO_2_/H_2_O, CH_3_CN and SO_2_ [9,20–26]. The aim of their work was to understand the thermodynamics and kinetics of chemical reactions in SCFs, then use SCF solutions for electrosynthetic purposes [19]. Initial work with NH_3_ showed through chronocoulometric techniques that the diffusivity and thus mass transport of *m*-chloronitrobenzene was enhanced in supercritical conditions [26]. Crooks & Bard [22,23] also reported on the first electrochemistry of organic compounds in scNH_3_; they noted that reactions in liquid NH_3_ could also be accomplished in scNH_3_. Their work demonstrated that the dielectric constant and density could be manipulated in scNH_3_ by changing the pressure or temperature [22]. Bard and co-workers [24,25,27] studied the use of H_2_O as an SCF for electrochemistry. The unique properties of water near its critical point render it a good solvent for both organics and salts [19]. The experimental difficulties associated with scH_2_O, including high critical temperatures, pressures (*T*_C_=647 K, *p*_C_=22.1 MPa) and corrosion, make it difficult to use [19,24]. McDonald *et al.* [24] described additional difficulties associated with working with scH_2_O including long equilibration times and the fact that electrodes could not be removed and replaced for cleaning or inspection; the latter is a common problem when working with any SCF.

Toghill *et al.* [28] have recently published a mini-review on SCFs that gives a brief overview of the literature relating to the electrochemistry in SCFs. Grinberg & Mazin [29] have reviewed the literature on electrochemistry in scCO_2_ and scCO_2_ with various co-solvents, up to 1998.

### (b) Electrochemistry in supercritical carbon dioxide

Although being dismissed as an SCF for preparative electrolyses by Silvestri *et al.* [18], carbon dioxide has been used as an SCF for electrochemistry [7,8,10,12–14,30–57]. Supercritical CO_2_, although having a low dielectric constant (*ε*<2) [31], is of interest as a solvent for electrochemistry as it is non-toxic, inexpensive, non-flammable and relatively inert. The easily accessible critical temperature and pressure (*T*_C_=304 K, *p*_C_=7.3 MPa) also make it attractive for electrochemical investigations [31]. The most extensive fundamental electrochemical studies of CO_2_ as an SCF have been performed by Abbott and co-workers. Abbott & Harper [31] carried out electrochemical investigations in scCO_2_ by dissolving hydrophobic electrolytes (tetrakis(decyl)ammonium tetraphenylborate) to give a conducting medium. They found some conductivity (approx. 10^−6^ S cm^−1^) and obtained some, poorly resolved, voltammetry [31]. Abbott & Harper [58] then used tetraalkylammonium tetraarylborates to make the first measurements of double-layer capacitance and conductivity in scCO_2_. In their work, they showed that tetraalkylammonium tetraarylborates in scCO_2_ led to an increased conductivity of the system. They also noted that high concentrations of the supporting electrolyte led to an increase in the viscosity of the supercritical media, thus small concentrations were used ranging from 0 to 0.03 mol dm^−3^.

The use of polar co-solvents has been investigated with the aim of raising the dielectric constant of scCO_2_. Goldfarb *et al.* [32] used varying amounts of methanol with scCO_2_ and studied the dielectric and voltammetric properties. Increasing the mole fraction of methanol resulted in a rise of the dielectric constant for the mixtures. The most extensive study of the use of co-solvents on the phase behaviour and conductivity of scCO_2_ has been reported by Bartlett *et al.* [47]. The investigation used both acetonitrile (CH_3_CN, *ε*=37) and methanol as potential co-solvents for scCO_2_. The high dielectric constant and complete miscibility with CO_2_ under easily accessible experimental conditions made both viable candidates [47]. Using a fixed molar ratio of co-solvent to CO_2_ (0.14), tetrabutylammonium tetrafluoroborate ([NBu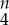][BF_4_]) was found to be five times more soluble (for similar pressures and temperatures) when acetonitrile was used as a co-solvent than methanol in scCO_2_ mixtures. The high solubility of the supporting electrolyte meant that acetonitrile was chosen as a co-solvent over methanol, although increasing the concentration of [NBu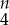][BF_4_] in scCO_2_/MeCN raises the pressure required to form a homogeneous solution.

**Figure 1.**
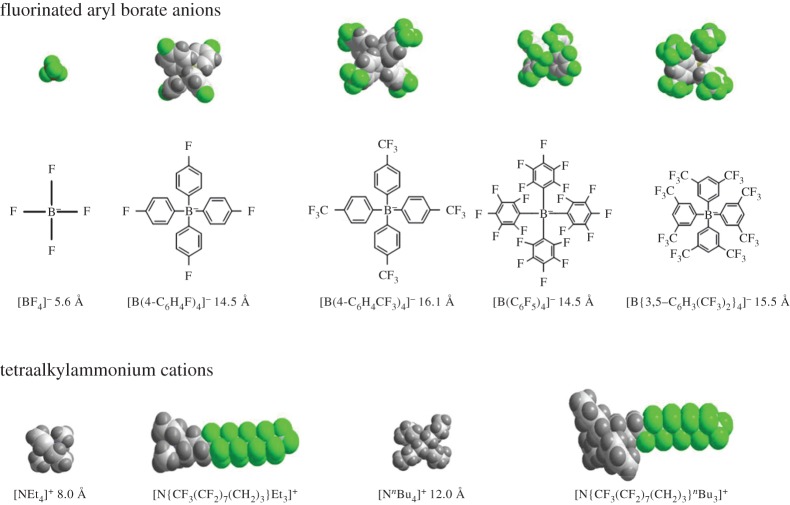
Space-filling representations of the cations and anions used as supporting electrolytes (green = F), illustrating their approximate sizes. The dimensions correspond to the estimated diameter across the respective ions based upon van der Waals radii [59]. (Reproduced with permission from PCCP Owner Societies.)

To enhance the conductivity of scCO_2_/MeCN mixtures, a range of electrolyte salts were synthesized (figure 1). The salts replaced the tetrabutylammonium cation or the tetrafluoroborate anion with varying derivatives. It was found that changing the anion from tetrafluoroborate to BARF (fluorinated derivatives of [BPh_4_]^−^) had the largest effect on the conductivity [47]. A tetrabutylammonium cation with a BARF anion ([NBu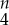][B(3,5-C_6_H_3_(CF_3_)_2_)_4_]^−^) was the most suitable supporting electrolyte. The electrolyte was found to have an order of magnitude higher molar conductivity than [NBu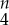][BF_4_]. Adding the fluorous ponytail to the cation in [N{CF_3_(CF_2_)_7_(CH_2_)_3_}Et_3_]^+^ and [N{CF_3_(CF_2_)_7_(CH_2_)_3_}^*n*^Bu_3_]^+^ has only a small effect, presumably because it does not significantly reduce the effect of ion-pairing.

### (c) The double layer in supercritical fluids

There has been very little work on the structure of the double layer at the electrode–SCF electrolyte interface, even though this determines the interfacial potential distribution and exerts a significant effect on the kinetics of electrode reactions.

Abbott & Eardley [60] have reported double-layer capacitance measurements in supercritical difluoromethane containing 20 mM tetrabutylammonium tetrafluoroborate at platinum elec- trodes at 363 K and three pressures. They concluded that at the lower pressures (13.5 and 16 MPa), where the dielectric constant for the solvent was lower (*ε*=8.5 and 8.1), the double-layer structure was described by the Helmholtz model whereas at higher pressure (26 MPa), where the dielectric constant was higher (*ε*=9.5), there was evidence for a contribution from the diffuse, or Gouy-Chapman, layer. Abbott & Harper [58] studied the double-layer capacitance for long chain quaternary ammonium electrolytes in supercritical CO_2_ under the low dielectric condition for scCO_2_ (*ε*=1.8); they found Helmholtz-like behaviour with the double-layer capacitance only very weakly dependent on the electrode potential.

**Figure 2.**
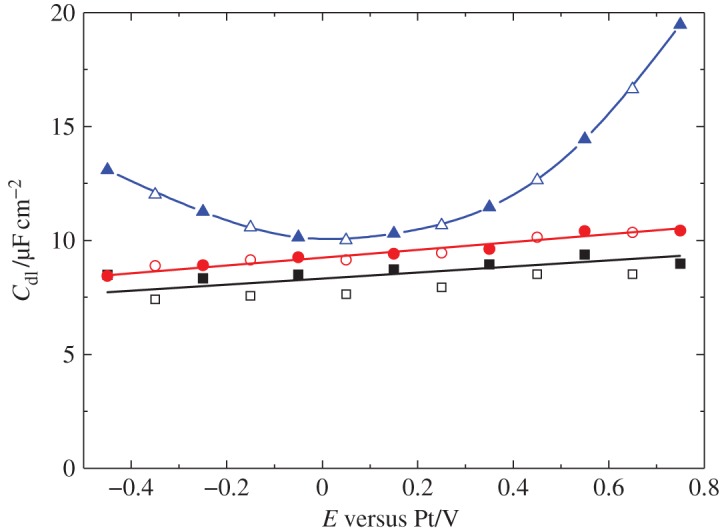
Comparison of *C*_dl_ for different substrates in scCO_2_/11 wt% CH_3_CN with 12.15 mM [NBu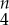][BF_4_] at 306 K and 15.5 MPa. Open symbols represent data taken on the cathodic scan; filled symbols on the anodic scan. Black squares: 0.5 mm diameter platinum disc; red circles: 0.5 mm diameter gold disc; blue triangles: 1 mm diameter glassy carbon disc [61]. (Reproduced with permission from [61]. Copyright XXXXXX Elsevier.)

Recently, Bartlett & Cook [61] published results for the study of the double-layer capacitance in scCO_2_/11 wt% MeCN mixtures of constant density between 306 K, 15.5 MPa and 316 K, 20.2 MPa. They found that the results for Pt and Au electrodes could be described by a simple Helmholtz layer model. In contrast, for glassy carbon electrodes, the results for the double-layer capacitance show a parabolic-like potential dependence attributed to the lower density of states near the Fermi level and the space charge contribution to the measured capacitance (figure 2).

### (d) Electrochemistry in supercritical hydrofluorocarbons

Another approach is to use HFCs as supercritical solvents as these are polar and give higher dielectric fluids while retaining reasonable critical temperatures and pressures. Fundamental studies of the conductivity of scHFCs with [NBu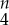][BF_4_] have been reported in the literature by Abbott and co-workers [62,63] and Olsen & Tallman [64]. Abbott and co-workers performed additional fundamental studies in scHFCs, which included the solubility of aromatic hydrocarbons [65], hydrogen bond interactions [16], electrochemical reduction of CO_2_ (performed in scCO_2_ with 1,1,1,2-tetrafluoroethane as a polar modifier) [33] and the effects of electrolyte concentration on viscosity and voltammetry [66].

Bartlett *et al.* [67] reported the phase behaviour and conductivity of electrolytes in scHFCs in a study analogous to their work in scCO_2_. HFCs have a higher dielectric constant than scCO_2_ and thus avoid the need for a polar co-solvent. The phase behaviour of trifluoromethane (CHF_3_), difluoromethane (CH_2_F_2_) and 1,1,1,2-tetrafluoroethane (CH_2_FCF_3_) containing [NBu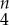][BF_4_], [NBu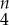][B(3,5-C_6_H_3_(CF_3_)_2_)_4_] and Na[B(3,5-C_6_H_3_(CF_3_)_2_)_4_] was studied and the conditions for forming a single supercritical phase were established. All three HFCs were found to be good solvents for [NBu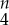][BF_4_], but the results showed that the CH_2_F_2_ system had the lowest *p*_C_ for dissolving a given amount of [NBu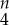][BF_4_]. Additionally, the solubility of Na[B(3,5-C_6_H_3_(CF_3_)_2_)_4_] in CH_2_F_2_ was found to be unexpectedly high. The conductivity of [NBu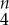][B(C_6_F_5_)_4_], [NBu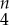][B(3,5-C_6_H_3_(CF_3_)_2_)_4_], [NR_*f*_Bu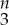][B(3,5-C_6_H_3_(CF_3_)_2_)_4_] (R_*f*_=(CH_2_)_3_C_7_F_15_) and Na[B(3,5-C_6_H_3_(CF_3_)_2_)_4_] was studied in scCH_2_F_2_. The results showed that these salts were more conducting than [NBu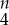][BF_4_] under the same conditions but the increase was much less significant than that reported for scCO_2_/MeCN [67]. It was concluded, however, that both the [NBu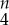][BF_4_] and BARF salts would be suitable background electrolytes for electrodeposition from scCH_2_F_2_.

### (e) Electrodeposition from supercritical fluids

Electrodeposition from SCFs is also sparse in the literature. The earliest work is that of Williams & Naiditch [68] in 1976 with Silvestri *et al.* [18] 5 years later. Williams & Naiditch [68] presented the deposition of silver from near- and supercritical NH_3_. In their work, Williams and Naiditch used silver nitrate (AgNO_3_) to electrodeposit silver in the form of needles and dendrites on a Pt cathode. AgNO_3_ was chosen due to its solubility in liquid NH_3_; additionally, electrodeposited Ag metal is insoluble in NH_3_. Although depositing Ag from the mixture, there is no identification as to the phase behaviour of the system. The temperature of the system is quoted by the authors but there is no indication of the pressure of the system. Having the system above a critical pressure and temperature is fundamental to being in the supercritical phase. Williams & Naiditch [68] concluded that electrodeposition had been performed in the supercritical phase. However, with no indication as to the pressure of the system, it is not clear that the system was indeed supercritical. Finally, McDonald *et al.* [24] in 1986 reported on the deposition of Cu from copper (I) chloride (CuCl_2_) under supercritical, or close to supercritical, H_2_O containing KCl at 573 K and 8.2 MPa.

Electrochemical syntheses of polymers from scCO_2_ and scCHF_3_ have also been reported [41,69]. Yan *et al.* [41] successfully electrochemically synthesized polypyrrole onto an indium tin oxide (ITO) electrode from a single-phase scCO_2_/MeCN mixture. The polypyrrole was found to form a smoother film than when deposited from acetonitrile. It was found that additional CO_2_ decreased the polymerization rate of the film in scCO_2_/MeCN, suggesting that the low viscosity of the sc system was not important in the growth rate of the smooth polypyrrole film. Atobe *et al.* [69] reported in 2004 the first successful electrochemical synthesis of polypyrrole and polythiophene from scCHF_3_ containing 10 mM pyrrole or thiophene with 40 mM [NBu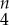][PF_6_] at 323 K and 15 MPa. Cyclic voltammetry performed in the system indicated that polymerization rates were increased in scCHF_3_ compared with MeCN [69]. It was reported that the films polymerized from scCHF_3_, although, being thinner than those produced in acetonitrile, they exhibited a higher electrochemical capacity (approx. 6× bigger for polypyrrole and approx. 10× higher for polythiophene). It was also observed that adjusting the pressure of scCHF_3_ resulted in switching the polymerization on or off [69]. This was attributed to the precipitation of [NBu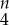][PF_6_] at lower pressures.

SCFs combine the properties of liquid and gas, and the lack of surface tension allows full penetration of high aspect ratio nanopores [8]. The first demonstration of electrodeposition into nanoporous templates was performed by Ke *et al.* [8] in 2009.Their work reported the deposition of 3 nm diameter copper nanowires in mesoporous silica templates on ITO. Depositions were carried using specially selected metal precursors, [Cu(MeCN)_4_][B(3,5-C_6_H_3_(CF_3_)_2_)_4_], and supporting electrolyte, [NBu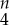][B(3,5-C_6_H_3_(CF_3_)_2_)_4_], in scCO_2_/MeCN (12.1 wt%) at 311 K and 17.2 MPa [8]. Ke *et al.* [8] also demonstrated the deposition of copper (from [Cu(MeCN)_4_][BF_4_]), silver ([Ag(MeCN)_4_][BF_4_]) and cobalt ([Co(Me(CN)_6_)][BF_4_]_2_) in scCO_2_/MeCN (13 wt%) using [NBu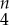][BF_4_] as a supporting electrolyte onto both micro- and macro-electrodes [8].

**Figure 3.**
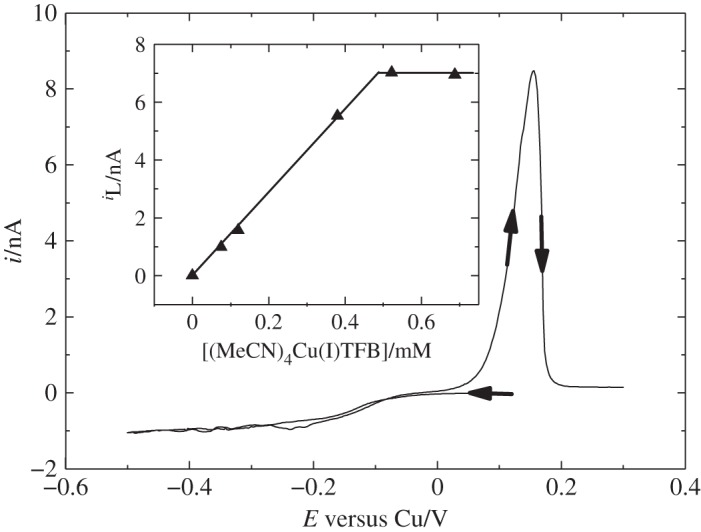
Copper voltammetry performed in scCO_2_ with 12.1 wt % CH_3_CN and [N^*n*^Bu][BF_4_] (20 mM) at 310 K and 17.24 MPa. Electrodes were: 25 μm diameter platinum disc working electrode, 0.5 mm diameter platinum disc pseudo reference and 0.5 mm diameter platinum wire counter electrode. The sweep rate was 20 mV s^−1^. The insert shows the limiting current as a function of the concentration of the copper complex. (Adapted from [44].)

In 2010, Cook *et al.* [44] reported the deposition of copper from two precursors, [Cu(II)(hfac)_2_] (where hfac is hexafluoroacetylacetonate) and [Cu(I)(MeCN)_4_][BF_4_], in scCO_2_/MeCN and supercritical trifluoromethane (CHF_3_) at 310–311 K and 17–20 MPa using either [NBu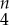][BF_4_] or [NBu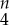][B(3,5-C_6_H_3_(CF_3_)_2_)_4_] as the supporting electrolyte. A disadvantage of using the Cu(II) salt is that the Cu(II) complex is quite oxidizing and can react with the deposited copper film, stripping it from the electrode as soluble Cu(I). Investigations with [Cu(MeCN)_4_][BF_4_] in scCO_2_/MeCN showed copper reduction with a single wave which reached a plateau along with a clear stripping peak on the reverse anodic scan, indicating that it was a more suitable complex for electrodeposition. To determine the solubility of [Cu(MeCN)_4_][BF_4_] in scCO_2_/MeCN, a series of microelectrode experiments were carried out with varying concentrations of the copper (I) complex. It was observed that the limiting current increased linearly at low concentrations and then reached a plateau at higher concentrations, leading to a solubility of 0.49 mM (figure 3). The diffusion coefficient for [Cu(MeCN)_4_][BF_4_] in scCO_2_/MeCN containing 20 mM [NBu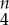][BF_4_] at 310 K was calculated to be 2.30×10^−5^ cm^2^ s^−1^; only slightly higher than the corresponding value in liquid MeCN at 296 K. Changing the counter-anion from [BF_4_] to [B(3,5-C_6_H_3_(CF_3_)_2_)_4_] allowed for a higher concentration to be used due to its greater solubility. Voltammetry performed showed identical features to those obtained with [NBu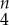][BF_4_], with an increased solubility up to at least 16 mM. The diffusion coefficient for [Cu(MeCN)_4_]^+^ in scCO_2_ with 12.1 wt% MeCN and 20 mM [NBu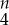][B(3,5-C_6_H_3_(CF_3_)_2_)_4_] at 310–311 K was calculated to be 3.30×10^−5^ cm^2^ s^−1^, which is approximately 1.5 times larger than the corresponding value in scCO_2_/MeCN with 20 mM [NBu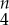][BF_4_].

In 2012, Ke *et al.* [70] reported the electrodeposition of germanium from both scCO_2_/MeCN and supercritical difluoromethane (scCH_2_F_2_). The electrochemistry and electrodeposition of [NBu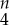][GeCl_3_] was studied both in liquid MeCN and in scCO_2_/MeCN. In scCO_2_/MeCN, amorphous deposits were produced on platinum and silicon working electrodes at low overpotentials (−1.4 V and 0.9 V versus Pt) with long plating times which produced only thin films. The films deposited in the supercritical phase contained very high levels of impurities of oxygen and carbon compared with liquid MeCN along with additional contamination from chlorine, fluorine and iron (which was attributed to chemical attack of the steel vessel of the reactor). Ke *et al.* [70] further investigated Ge (IV) reagents in both scCO_2_/MeCN and scCH_2_F_2_. GeCl_4_ was investigated as a Ge (IV) reagent; it was also found that GeCl_4_ was more stable at higher temperatures, providing a wider operating temperature range for electrodeposition. In scCH_2_F_2_, an initial reduction peak was observed at −0.7 V versus Pt followed by a less resolved increase in cathodic current at −1.5 V versus Pt, which was presumed to be the two-step reduction of GeCl_4_ observed in liquid CH_2_F_2_. Energy dispersive X-ray analysis confirmed the presence of Ge and Raman spectroscopy confirmed that amorphous elementary Ge was electrodeposited from scCH_2_F_2_.

**Figure 4.**
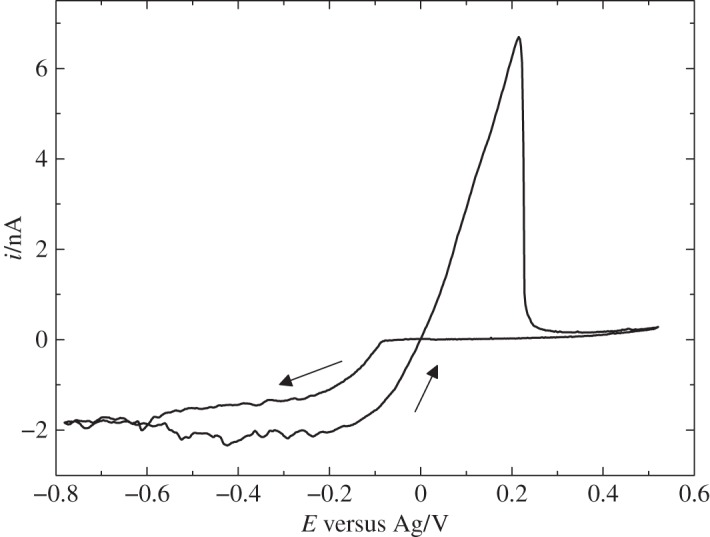
Cyclic voltammetry in 0.09 mM [Ag(CH_3_CN)_4_][BF_4_] in 20 mM [^*n*^Bu_4_N][BF_4_] scCO_2_/CH_3_CN (approx. 15% v/v) at 310 K and 172 bar, at a 51 μm Pt disc, recorded at 0.02 V s^−1^. (Adapted from [46].)

Bartlett *et al.* [46] reported on the electrodeposition of silver from scCO_2_/MeCN. In the study, five silver precursors were investigated. For the precursor, [Ag(CH_3_CN)_4_][BF_4_], typical cathodic metal deposition and anodic stripping were observed; fluctuations are seen on the steady-state current and these are attributed to the convection in the cell caused by temperature gradients and are exacerbated by the low viscosity of the medium (figure 4).

A comprehensive review of electrodeposition from SCFs can be found in the recent perspective article by Bartlett *et al.* [59].

## 3. Electrochemistry of metallocenes in supercritical fluids

### (a) Metallocenes as redox standards in non-aqueous solutions

Conventional reference electrodes, such as the saturated calomel electrode, are unsuitable for use in SCFs and for this reason it is desirable to have some suitable internal reference standard that can be used in combination with a Pt *pseudo*(or *quasi*) reference electrode for experimental measurements. In the case of non-aqueous solutions, the International Union of Pure and Applied Chemistry (IUPAC) recommends the use of the ferrocene/ferrocenium (Fc/Fc^+^) redox couple [71–73]. In 1999, Noviandri *et al.* [74] questioned the use of the ferrocene/ferrocenium redox couple as an internal standard as studies had shown that in some media there was a tendency for the Fc^+^ ion to react with nucleophiles. Bashkin & Kinlen [73] have proposed the use of decamethylferrocene/decamethylferrocenium (DMFc/DMFc^+^) as an internal reference standard on the basis of its stability to molecular oxygen and other factors. In this case, the bulky methyl groups of the DMFc/DMFc^+^ are expected to reduce specific and non-specific interactions between the redox couple and the solvent molecules [74].

The electrochemistry of DMFc/DMFc^+^ in non-aqueous solvents has been studied by a number of groups under a variety of conditions and the couple exhibits a reversible one-electron redox process at the electrode with facile electron transfer kinetics [9,73–79]. Noviandri *et al.* [74] extensively studied the solvent dependencies of the formal redox potential of the Fc/Fc^+^, DMFc/DMFc^+^ and 1,2,3,4,5-pentamethyl-ferrocene (Me_5_Fc) couples in 29 solvents including water. They concluded that DMFc/DMFc^+^ was a more suitable redox standard than Fc/Fc^+^ [74]. They also concluded that DMFc/DMFc^+^ complied with all of the IUPAC requirements for a reference redox standard and was superior to the Fc/Fc^+^ redox system for studying solvent effects on the thermodynamics of electron transfer of other couples.

The cobaltocenium (CoCp^+^/CoCp_2_) redox couple should give a formal redox potential that is sufficiently separated from the DMFc/DMFc^+^ to give a half-wave potential separation which is constant and solvent independent. Stojanovic & Bond [80] examined the CoCp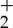/CoCp_2_ couple with respect to the Fc/Fc^+^ couple and found that the half-wave potential separation calculated was essentially independent of the electrode material, electrolyte and solvent used [80]. Aranzaes *et al.* [81] made measurements of the redox potential (*E*_1/2_) of a variety of metallocenes (including decamethylferrocene and decamethylcobaltocene) in a range of solvents (including acetonitrile and dichloromethane). They found that the difference in *E*_1/2_ values for decamethylferrocene and decamethylcobaltocene was solvent independent [81].

### (b) Metallocenes as redox probes in supercritical fluids

In 1988, Crooks & Bard [21] studied the electrochemistry of ferrocene and phenazine in near- and supercritical acetonitrile between 298 and 573 K. It was found that the one-electron oxidation wave of ferrocene at low scan rates was reversible or nearly reversible throughout the entire temperature range studied. Due to the high temperatures employed, tetraalkylammonium salts could not be used as they are not thermally stable; therefore, CF_3_SO_3_Na was used as the supporting electrolyte. When CF_3_SO_3_Na was used as a supporting electrolyte in scNH_3_, the resistance of the solution increased with increasing temperature; however, in acetonitrile, the conductivity of the system approximately doubled as the temperature was increased from 298 to 373 K and continued to rise up to 573 K. At subcritical temperatures, an uncomplicated one-electron oxidation of Fc to Fc^+^ was observed. Near theoretical peak-to-peak potential separation was seen at low scan rates; also, the peak potential was found to be independent of scan rate over four decades with the ratio of peak anodic and peak cathodic current being unity over 523 K. The diffusion coefficient of ferrocene was also measured at both 298 and 548 K. It was found that the diffusion coefficient increased from 2.6×10^−5^ cm^2^ s^−1^ at 298 K to 2.4×10^−4^ cm^2^ s^−1^ at 548 K (12.5 MPa). This order of magnitude increase in the diffusion coefficient was comparable to that measured for phenazine. It was also noted that ‘mild filming’ of the electrode occasionally occurred above *T*_C_, but when the solution was cooled no evidence for the filming was observed.

In 1989, Cabrera & Bard [9] studied the electrochemistry of various organometallic complexes, including decamethylferrocene, in near- and supercritical acetonitrile. Decamethylferrocene exhibited reversible or quasi-reversible behaviour from 298 to 555 K (supercritical region). As the temperature was increased the peak-to-peak potential separation increased; this was attributed to an increase in uncompensated resistance. To calculate the diffusion coefficient of decamethylferrocene in the system, Cabrera and Bard used the peak anodic current and assumed a reversible wave at the given temperature. It was found that the diffusion coefficient increased from 1.8×10^−5^ cm^2^ s^−1^ at 298 K (0.1 MPa) to 9.3×10^−5^ cm^2^ s^−1^ at 521 K (11.6 MPa). This large increase in *D* parallels Fc in MeCN (as described above) and reflects the strong decrease in viscosity of the solution at higher temperatures.

In 1994, Olsen & Tallman [82] studied the voltammetry of ferrocene in sub- and supercritical chlorodifluoromethane (scCHClF_2_). Chlorodifluoromethane has a critical temperature and pressure of *T*_C_=369.15 K and *p*_C_=4.97 MPa, along with a higher dielectric constant (*ε*=2.31 at the critical point) than carbon dioxide (*ε*=1.18 at the critical point). Furthermore, the more polar solvent permits the dissolution of a small quantity of electrolyte, making voltammetric measurements at microelectrodes possible with minimal Ohmic distortion [82]. Electrochemistry of ferrocene was obtained at three conditions: normal liquid conditions (299 K, 5.2 MPa), near critical and supercritical (378 K, 15 MPa) conditions. Although it was possible to obtain voltammetry near the critical point, it was not possible to obtain reproducible electrochemistry. This is attributed to the changes in fluid composition with respect to the amount of electrolyte added, making the precise control of the density and dielectric constant extremely difficult [82]. Cyclic voltammetry of ferrocene performed in the supercritical phase showed a typical microelectrode response and was analogous to that performed at 299 K, although the similar limiting current recorded for both conditions was ascribed to some passivation of the electrode surface. The diffusion coefficient of ferrocene was determined for both liquid and supercritical conditions and showed an increase from 2.36×10^−5^ cm^2^ s^−1^ (298 K, 5.20 MPa) to 1.30×10^−4^ cm^2^ s^−1^ (388 K, 9.00 MPa); this order of magnitude increase is consistent with that found by Crooks & Bard [21].

Olsen & Tallman [82] also demonstrated the reduction of cobaltocenium hexafluorophosphate to cobaltocene. The steady-state voltammogram displayed nearly reversible behaviour from Nernstian analysis. Voltammetry was also performed in liquid CHClF_2_ containing both ferrocene and cobaltocenium; the half-wave potential separation of 1.33 V was in good agreement with literature value and further tests were to be performed in supercritical conditions.

In 1996, Olsen & Tallman [64] reported further on the conductivity and voltammetry of ferrocene and cobaltocenium in liquid and scCHClF_2_. Voltammetry of both ferrocene and cobaltocenium at equal concentration in liquid conditions (298 K, 5.20 MPa) show Nernstian behaviour. At 298 K, the half-wave potential difference was found to be 1.32 V and is again in good agreement with the literature. The hydrodynamic radii of both ferrocene and cobaltocenium in both acetonitrile and dichloromethane were estimated from the Stokes–Einstein equation. Ferrocene has an estimated hydrodynamic radius of 2.8 Åin acetonitrile and 4.0 Åin dichloromethane; cobaltocenium has an estimated hydrodynamic radius of 3.5 Å in acetonitrile and 5.5 Å in dichloromethane. Voltammetry of the metallocenes performed in scCHClF_2_ at varying pressures (12, 20 and 30 MPa) showed two interesting changes in the responses for ferrocene oxidation and cobaltocenium reduction; one is that both waves were shifted to more negative potentials but not to the same magnitude, the second is that the limiting current for ferrocene decreased with pressure but those for cobaltocenium remained constant. The potential of the quasi-reference is likely to be a function of pressure but should shift each potential wave by identical amounts and not have an effect on Δ*E*_1/2_: although the shift is small (approx. 40 mV) the behaviour was noted as reproducible. The variation in Δ*E*_1/2_ with pressure was attributed to the variation in fluid resistance with pressure and the variation in ohmic distortion of the voltammograms [64]. Analysis of the limiting current for ferrocene at increasing pressure showed a decrease, whereas cobaltocenium remained constant. The diffusion coefficient was measured for multiple concentrations of ferrocene at the same pressure; the diffusion coefficient was shown to decrease rapidly with pressure, which is the result of a rapid increase in density and viscosity with pressure. Voltammetry of ferrocene was also performed in supercritical trifluoromethane (CHF_3_) where reversible Nernstian behaviour was observed.

In 2000, Goldfarb & Corti [83] studied the electrochemistry of decamethylferrocene and decamethylferrocenium hexafluorophosphate (DMFc^+^) in supercritical trifluoromethane. In the study, linear sweep voltammetry was performed at a platinum microelectrode (25 μm Ø) on both DMFc and DMFc^+^ at 323.15 K at several densities with and without supporting electrolyte (tetrabutylammonium hexafluorophosphate). It was found that, even in the complete absence of supporting electrolyte, the shape of the voltammogram (for DMFc) was still preserved, and despite the large Ohmic drop the analysis of the limiting current obtained from the curve did not lose accuracy. A plot of *E* versus log[(*I*_L_−*I*)/*I*] was performed for DMFc and gave a value of 64.1 mV for the exchange of a single e^−^ under reversible conditions at 323.15 K; in excess of supporting electrolyte the value was as high as 192 mV. This is attributed to the lower dielectric constant of the solvent at lower densities, leading to a higher degree of association and consequently less free ions from the supporting electrolyte. In the absence of supporting electrolyte, impurities from the solvent contribute to some extent to the conductivity of the medium. In contrast, it was found that DMFc^+^ is strongly influenced by the concentration of supporting electrolyte. The decrease in concentration of supporting electrolyte increases the limiting current of DMFc^+^; this is due to the increases in the migrational current.

In 2004, Goldfarb & Corti [84] further studied the diffusion of decamethylferrocene and decamethylferrocenium hexafluorophosphate in supercritical trifluoromethane. The diffusion coefficients of DMFc and DMFc^+^ in scCHF_3_ were measured at a temperature of 323.15 K as a function of density, with TBAPF_6_ as the supporting electrolyte on a platinum microelectrode. For DMFc^+^, the temperature of the system and concentration of DMFc^+^ and TBAPF_6_ were kept constant and the pressure of the system was altered to give a different density and thus a varying viscosity [84]. At 323.15 K and a concentration of *c*_DMFc+_=1×10^−6^ mol dm^−3^, the pressure was varied from 7.67 to 15.73 MPa to give a density range from 0.4971 to 0.9058 g cm^−3^ and a viscosity of 3.23 to 6.88×10^−5^ Pa s [84]. This increase in density decreases the diffusion coefficient from 12.3×10^−5^ to 7.1×10^−5^ cm^2^ s^−1^, as indicated by the decrease in the limiting current from 60 to 35 pA, and the trend is repeated for lower and higher concentrations of DMFc^+^ and when the temperature of the system is lowered. The same study was performed on DMFc at 323.15 K and *c*_DMFc_=4.83×10^−6^ mol dm^−3^; the pressure was varied from 8.45 to 15.66 MPa to give a density range from 0.6011 to 0.9045 g cm^−3^ and a viscosity of 3.90 to 6.86×10^−5^ Pa s. The increase in density again decreases the diffusion coefficient from 13.0 to 7.3×10^−5^ cm^2^ s^−1^ and the limiting current decreases from 292 to 171 pA; again, this trend continues for varying temperatures and concentrations.

In 2013, Toghill *et al.* [45] studied the voltammetry of decamethylferrocene in a supercritical carbon dioxide medium (containing acetonitrile). In their experiments, they used scCO_2_ with up to 0.41 mole fraction MeCN and tetradecylammounium tetrakis(pentafluorophenyl)borate (TDATFPB; a room temperature ionic liquid) as the supporting electrolyte. Cyclic voltammetry performed with 0.5 mM DMFc and 10 mM TDATFPB in scCO_2_/MeCN (0.41 mole fraction) at 100 bar and 313 K showed a redox process occurring with the typical peak shape (above 25 mV s^−1^) attributed to semi-infinite linear diffusion. They attributed this to the presence of an approximately 60-μm-thick, liquid-like layer at the electrode surface.

**Figure 5.**
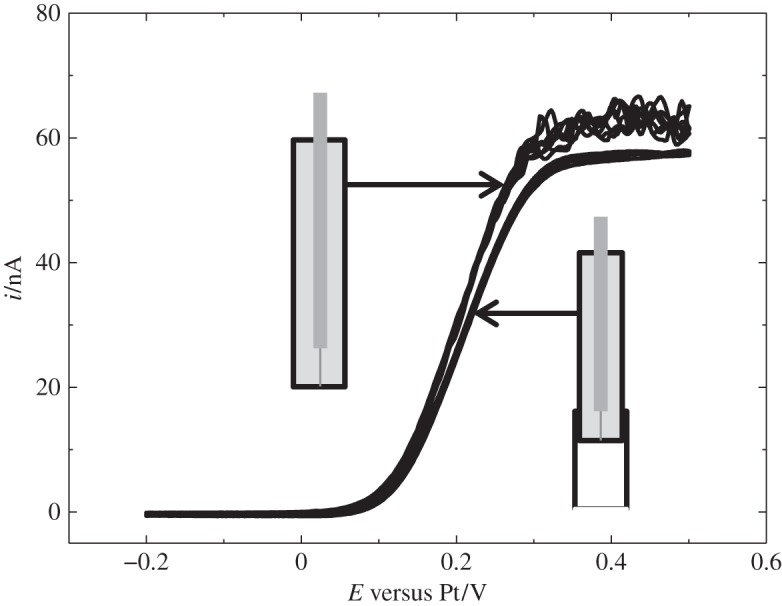
Cyclic voltammetry in 1.42 mM DMFc in supercritical CO_2_ with 15 wt% MeCN containing a 20 mM [NBu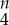][BF_4_] supporting electrolyte at 309 K. The working electrodes were both nominally 50 μm diameter platinum discs with and without a baffle; the counter and reference electrode were 0.5 mm diameter platinum wires. *p*=17.58 MPa; *p*=17.49 MPa, respectively. Sweep rate 10 mV s^−1^ for all electrodes [85]. (Reproduced with permission from PCCP Owner Societies.)

Branch *et al.* [85] carried out detailed studies of the voltammetry of decamethylferrocene in scCO_2_/MeCN (15 wt%), 20 mM [NBu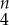][BF_4_] at 309 K and 17.5 MPa at Pt microdisc electrodes of three different sizes. They found that, on Pt, a passivating film could be formed but that if this was removed by cathodic cycling, the voltammetry was well behaved. There was no evidence for the formation of this type of film on gold macro-electrodes under the same conditions. In all cases, the voltammetry showed significant noise under mass-transport-limited conditions caused by natural convection within the cell and they were able to significantly reduce this by placing a baffle around the microelectrode (figure 5). For the platinum microelectrode, they found reversible 1e^−^ redox behaviour for decamethylferrocene with a formal potential of 0.115 versus Pt pseudo reference electrode. From the microdisc electrode measurements, they obtained a value for the diffusion coefficient of DMFc of *D*=4.06×10^−5^ cm^2^ s^−1^ in scCO_2_/MeCN (15 wt%), 20 mM [NBu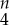][BF_4_] at 36°C and 17.5 MPa.

## 4. Conclusion

In this review, we have given an overview of electrochemistry in SCFs from the earliest studies to the current state of the art. We have highlighted key results in SCF electrochemistry including double-layer capacitance studies, electrodeposition of 3 nm diameter nanowires, and the use of metallocenes as redox probes and standards. We have shown how carbon dioxide is used as an electrolyte for electrochemistry by the addition of a polar co-solvent (such as acetonitrile) along with variations of tetraalkylammonium tetraarylborate supporting electrolytes. We have also discussed how HFCs, through their polar nature and higher dielectric constant, can be used as supercritical solvents without the addition of a co-solvent. Notwithstanding the recent advances in the use of SCFs as media for electrochemistry, SCF electrochemistry remains relatively little studied. However, despite this, there are clear benefits to using SCFs for electrochemistry, particularly in the area of nanomaterial deposition.
